# Eigen damping constant of spin waves in ferromagnetic nanostructure

**DOI:** 10.1038/s41598-019-49872-w

**Published:** 2019-09-13

**Authors:** Indra Purnama, Jung-Hwan Moon, Chun-Yeol You

**Affiliations:** 1 Department of Emerging Materials Science, DGIST, Daegu, 42988 South Korea; 2 Department of Materials Science and Engineering, Korea University, Seoul, 02841 Korea; 3 Advanced Technology Development Team, Semiconductor R&D Center, Samsung Electronics Co., Ltd., Gyeonggi-Do, 445-701 Korea; 4 Global Center for Bio-Convergence Spin System, DGIST, Daegu, 42988 Korea

**Keywords:** Spintronics, Ferromagnetism

## Abstract

Though varying in nature, all waves share traits in a way that they all follow the superposition principle while also experiencing attenuation as they propagate in space. And thus it is more than common that a comprehensive investigation of one type of wave leads to a discovery that can be extended to all kinds of waves in other fields of research. In the field of magnetism, the wave of interest corresponds to the spin wave (SW). Specifically, there has been a push to use SWs as the next information carriers similar to how electromagnetic waves are used in photonics. At present, the biggest impediment in making SW-based device to be widely adapted is the fact that the SW experiences large attenuation due to the large damping constant. Here, we developed a method to find the SW eigenmodes and show that their respective *eigen damping constants* can be 40% smaller than the typical material damping constant. From a bigger perspective, this finding means that the attenuation of SW and also other types of waves in general is no more constrained by the material parameters, and it can be controlled by the shape of the waves instead.

## Introduction

In nature, one way for energy to be transferred from one position in space to another position is via a wave. Although the method of generation as well as propagation may differ between one type of wave to the others, once generated, a wave will travel to its surrounding without further need of additional external energy source. This fact has made the use of waves to be attractive as information carrier, such as the use of electromagnetic waves in telecommunication. In the field of magnetism, the wave of interest corresponds to the spin wave (SW). As the transmission of SWs in a magnetic material does not necessarily require the application of electrical current, SW-based devices have been envisioned to require much less energy to operate as compared to their electronics counterparts due to negligible Joule heating. Already, huge progress has been made to realize SW-based device for real-life applications, such as the development of SW transistor and multiplexer for logic applications^1–11^. However, one concern with using ferromagnetic material as the medium for SW device is the high damping constant^12^, which results in the SW losing significant amplitude and energy during its propagation.

It is long believed that the damping is purely determined by the materials parameters and the ellipticity of the ferromagnetic resonance (FMR) precision^13–15^. Due to this, yttrium iron garnet (YIG) have been considered as a potential medium for SW devices^16–23^ for its very low damping constant which allows the SWs to propagate in a much longer distance. Nevertheless, YIG is not compatible with the current silicon integrated circuit technology, as it requires the use of gadolinium gallium garnet (GGG) substrate for its growth. Therefore, efforts to optimize SW transmission in ferromagnetic nanostructures are still undertaken, such as by engineering the layer structure^24,25^, annealing^26^, interface control^27,28^, fabrication conditions^29^, etc. However, it has also been shown that different SW shapes experience different damping^30–32^, which opens up new possibility of reducing the damping constant by engineering the shape of the injected SW.

In this work, we report on a method to find the SWs shapes with the lowest damping constant in patterned magnetic nanostructures, i.e. the SW eigenmodes. Subsequently, we show that the eigenmodes have unique damping constants of their own, which we call them *eigen damping constant*. To illustrate our method, we primarily focus on the case of SW generation and detection in a magnetic nanowire with in-plane magnetization anisotropy as a simple example; however, the method can be applied to any kind of magnetic system. We show that the damping constant of each SW eigenmode has different value and they can be even 40% smaller than the original material damping constant, which leads to a higher SW transmission. Due to the fact that the transmission enhancement comes from the engineering of the wave shape of the SW itself, this means that our method is extendable to other branch of wave physics as it comes from the interference capabilities of waves which is universal.

## Methods

### Micromagnetic simulation

The simulations were performed using Mumax3 micromagnetic simulator^33^ to generate SWs in a nanowire with an in-plane magnetization. For simulations of nanowires with in-plane magnetization, the material parameters were chosen to correspond to Ni_80_Fe_20_, i.e. saturation magnetization *M*_*s*_ = 860 × 10^3^ A/m, exchange stiffness *A*_*ex*_ = 1.3 × 10^−11^ J/m, and Gilbert damping *α* = 0.02^34^. The nanowire had a length of 2400 nm and a thickness of *d* = 5 nm while the width, *w*, was varied from 30 nm to 500 nm. The simulation cell size was 5 nm × 5 nm × 5 nm, which is the typical cell size for this setup^35^. The magnetization direction of the nanowire was directed along the length of the wire. The time step of the simulation was 1 ps to ensure the accuracy of the following matrix calculation. Simulation results with lower cell size, thicker nanowires, and smaller *α* are discussed in Supplementary Material III.

## Results

### Transmission matrix of spinwave

**Figure 1:**
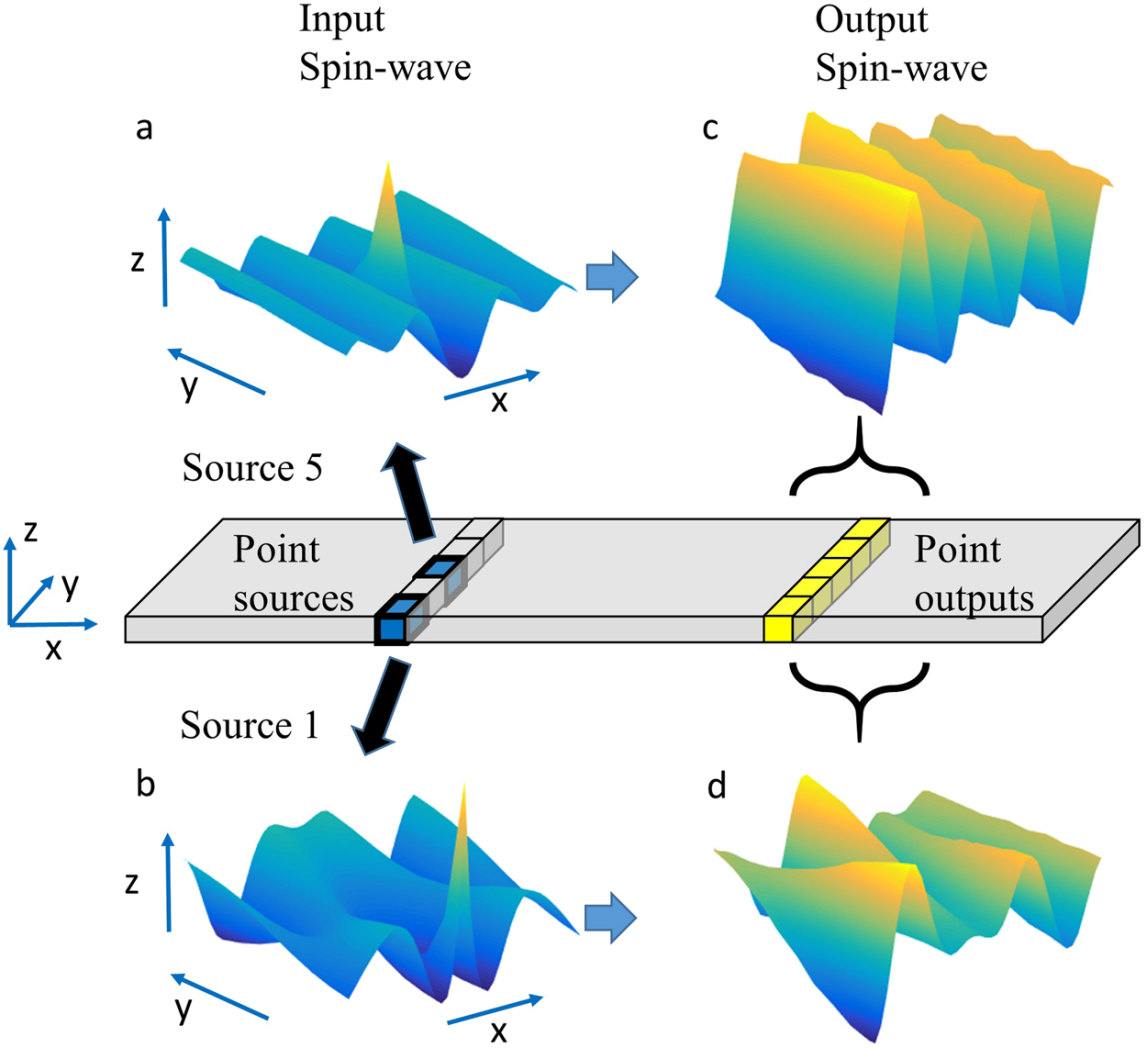
Schematic of the transmission matrix building method. Schematic showing how the input SW is divided into several point-source waves. The red lines represent the location of the point-sources, while the green line represents the location of the point-outputs. (**a**,**b**) The SWs that are observed at the point-sources when point-source 5 and point-source 1 are excited, respectively. Point-source 5 is located at the middle of the nanowire while point-source 1 is located at one edge of the nanowire. (**c**,**d**) The SWs that are observed at the point-outputs when point-source 5 and point-source 1 are excited, respectively. Amplitude of the waves in the image are exaggerated for easier visualization and understanding.

Here, the transmission of a SW from one point to the other is represented by an element of transmission matrix, which was constructed in the linear response regime and used to determine the eigenvalues, the eigenmodes, as well as the eigen damping of the system. In general, the transmission matrix concepts have been widely used in many field of physics, including transport theory^36^, electromagnetic waves theory^37^, color image processing^38^, and so on. The transmission matrix connects the output SW with the input SW in a simple form of $Y=T\cdot X$, where *Y*, *T*, and *X* are the output, the transmission matrix, and the input, respectively. In order to create the transmission matrix, first we assume that any input wave is a sum of smaller waves created by ‘point-sources’ across the width of the nanowire (Fig. 1). This assumption holds as we are working within the linear regime, where the superposition principle of waves holds true. Similarly, the output wave at the observation point is also considered to be composed of smaller waves that go to ‘point-outputs’. To illustrate our method, we first performed micromagnetic simulations with Mumax3^33^ on a nanowire with in-plane magnetization.

The transmission matrix building process is started by creating point-source excitations (Fig. 1). Each cell across the center of the nanowire is designated as a point-source, where we proceeded to generate a SW in each of them. The sources are labelled according to their position (e.g. Source 1, Source 2, …., and Source *N*). Here, *N* denotes the total number of point-sources. In our method, every simulation cell along the width of the nanowire is regarded as a point-source, thus *N* = 10 for a nanowire with a width (*w*) of 50 nm while *N* = 40 for a nanowire with a width of 200 nm and so on. The point-sources were excited with a localized excitation field of ${H}_{ext}={H}_{0}\,\sin \,2\pi ft$ along the *z* direction, where the excitation frequency *f* was varied across the simulations while the field amplitude was kept constant at *H*_0_ = 100 mT. The individual point source excitations create different SWs in the nanowire. Exciting point source 5 creates a relatively even SW (Fig. 1a) while exciting point source 1 creates an uneven SW profile across the width of the nanowire (Fig. 1b). Note that even when the external field was only excited at Source 1, in steady-state situation, SWs would be observed at all point-sources because all cells are exchange coupled to each other. The different SW created at the point-sources invoke different SW reading at the point-outputs (Fig. 1c,d). Here we observed the output SW at a distance of *l* = 250 nm away from the SW generator. The results show that the SWs at each of the point-sources and the point-outputs are not identical, which makes the relations between them non-trivial. In general, any input *X*(*t*) SW can be expressed by:

1 $$X(t)={X}_{1}(t)+{X}_{2}(t)+\cdots \,{X}_{3}(t)$$

where *X*_*i*_ represents a point excitation at Source *i*. However, (Fig. 1a,b) point-source excitation creates SWs at other sources at steady state. Therefore, we can write each *X*_*i*_ as a matrix:

2 $${X}_{i}(t)=(\begin{array}{c}{X}_{1i}(t)\\ {X}_{2i}(t)\\ \begin{array}{c}\vdots \\ {X}_{Ni}(t)\end{array}\end{array})$$

where *X*_*ji*_ represents the SW that is observed at point-source *j* due to a field excitation at point-source *i*. We can then represent *X*(*t*) as a whole by augmenting the above matrix to an *N* × *N* matrix that gives the information of how the point-sources respond to the field excitation *H*_*ext*_:

3 $${X}_{i}(t)=(\begin{array}{ccc}{X}_{1i}(t) & \cdots  & {X}_{1N}(t)\\ \vdots  & \ddots  & \vdots \\ {X}_{Ni}(t) & \cdots  & {X}_{NN}(t)\end{array})$$

Additionally, we can also define a susceptibility matrix χ that relates *X*(*t*) and the excitation field as follows:

4 $${X}_{i}(t)=(\begin{array}{c}{X}_{1i}(t)\\ {X}_{2i}(t)\\ \begin{array}{c}\vdots \\ {X}_{Ni}(t)\end{array}\end{array})=(\begin{array}{ccc}{\chi }_{1i}(t) & \cdots  & {\chi }_{1N}(t)\\ \vdots  & \ddots  & \vdots \\ {\chi }_{Ni}(t) & \cdots  & {\chi }_{NN}(t)\end{array})(\begin{array}{c}{H}_{1}(t)\\ {H}_{2}(t)\\ \begin{array}{c}\vdots \\ {H}_{N}(t)\end{array}\end{array})$$

where *H*_*i*_ = (*H*_0_ sin(*2πft*))_*i*_ is the localized excitation field at *i*-th point-source. Similarly, we can represent the output *Y*(*l*, *t*) in our nanowire as an *N* × *N* matrix that gives the information of how the input SW has changed as it arrives on the output location (*l*):

5 $${X}_{i}(t)=(\begin{array}{ccc}{X}_{1i}(t) & \cdots  & {X}_{1N}(t)\\ \vdots  & \ddots  & \vdots \\ {X}_{Ni}(t) & \cdots  & {X}_{NN}(t)\end{array})$$

In this way, each of the output waves *Y*_*i*_(*l*, *t*) and the input waves *X*_*i*_(*t*) are related by:

6 $${Y}_{i}(l,t)=T(l)\cdot {X}_{i}(t),$$

and subsequently:

7 $$Y(l,t)=T(l)\cdot X(t)$$

where *T*(*l*) is a *N* × *N* square matrix that correlates the waves of the point-outputs and the waves of the point-sources, with *l* being the position of the output on the nanowire relative to the input. Note that both *X* and *Y* are obtained from the oscillation of the magnetization The transmission matrix *T* can then be obtained by performing:

8 $$T(l)=Y(l,t)\cdot {(X(t))}^{-1}$$

(An example of *T* is shown in Supplementary Material I). By knowing *T*, it is then possible for us to find the SW eigenvalues and eigenmodes of the given nanowire at a given excitation frequency and location. Let us introduce a diagonal matrix which consists of eigenvalues $\Lambda ={m}_{ij}={m}_{i}{\delta }_{ij}$; here *m*_*i*_ is *i*-th eigenvalue and *δ*_*ij*_ is the Kronecker delta. We also introduce a matrix *Q* whose columns are *q*_*i*_: the *i*-th eigenmode. We then have the well-known relations, $T=Q\Lambda {Q}^{-1}$ and $T{q}_{i}={m}_{i}{q}_{i}$ by definition of eigenvalues and eigenmodes. From the definition of the transmission matrix, the output of *i*-th eigenmode input (if *X*(*t*) = *q*_*i*_) is simply written as:

9 $$Y(l,t)=T(l)X(t)=T(l){q}_{i}={m}_{i}{q}_{i}$$

The results show that there is simple multiplicity relation between the output and input: the output SW has the same wave form of the input SW, only the magnitude and phase are changed by factor of *m*_*i*_. (see the Supplementary Material II and Supplementary Videos Eigen0-3.avi). In general, the magnitudes of eigenvalues are smaller than 1, which represents the decay of the SW as it propagates within the medium.

Let us define the eigenvalue for a traveling distance of *Δl* as *m*_*i*_. The output SW at distance *Δl* for eigenmode *q*_*i*_ can be expressed by *Y*(*Δl*)* = m*_*i*_*q*_*i*_. Subsequently, due to the multiplicity relation, the SW at any position *l = nΔl* can then be expressed by:

10 $$Y(l)=Y(n\varDelta l)={{m}_{i}}^{n}{q}_{i}={{m}_{i}}^{\frac{l}{\Delta l}}{q}_{i}$$

Therefore, we can obtain the output SW at any *nΔl* position by using a known eigenvalue for the corresponding eigenmode.

**Figure 2:**
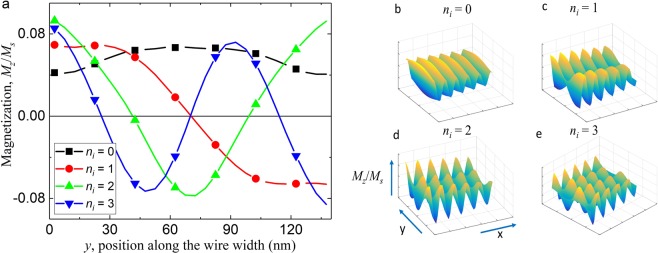
Magnetization configuration of the eigenmodes. (**a**) Plot showing the magnetization configuration across the wire width of the first four SW eigenmodes. The wire width here is 140 nm and the applied field strength is 100 mT with a frequency of 30 GHz. The magnetization configuration can be approximated by ${q}_{i}={A}_{i}\,\sin [\frac{({n}_{q}+1)\pi y}{{w}_{eff,i}}]$. (**b–e**) Visualization of the first four eigenmodes. The *x* and *y* axes are the physical axis of the nanowire while the vertical axis represents the magnetization oscillation of the SW.

As the eigenvalue represents the amplitude of the SW, for device application purpose, the most interesting eigenmode is the one with the highest eigenvalue. By employing our method to the data obtained from the micromagnetic simulations, we find that the eigenmodes of the ferromagnetic nanowire have standing wave-like form along the wire width with nodes and open boundary conditions (Fig. 2a). We number the eigenmodes by their number of nodes, *n*_*i*_. Where *n*_*i*_ = 0, the eigenmode is almost a plane wave without node, which we denote as *q*_0_. For the next eigenmode *q*_1_ with *n*_*i*_ = 1, the eigenmode has one node at the center of the wire, while eigenmode *q*_3_ has 3 nodes (Fig. 2b–e) and so on. The magnetization configuration of the eigenmodes can be approximated by:

11 $${q}_{i}={A}_{i}\,\sin [\frac{({n}_{i}+1)\pi y}{{w}_{eff,i}}]$$

where *A*_*i*_ is the amplitude of the eigenmode, *y* is the position coordinate along the width of the nanowire while ${w}_{eff,i}$ is the effective width of the nanowire that is used to take into account the soft pinning of the magnetizations at the edge of the nanowire^39^.

### Eigen damping constant of spinwave

**Figure 3:**
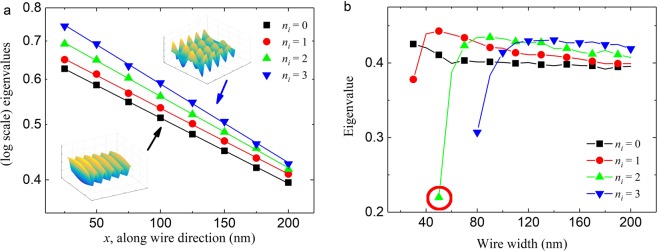
Eigenvalues of the eigenmodes. (**a**) Graph showing the eigenvalues in natural logarithmic scale of the first four modes as function of SW propagation distance in a nanowire with width of 140 nm. (**b**) The eigenvalues of the first four eigenmodes for different nanowire width. The frequency of the applied field was 30 GHz. The circled data points (red circle) show the eigenvalue of the *n*_*i*_ = 2 eigenmode in the first instance it is observed in the system.

The linear decay in log-scale (*y*-axis) eigenvalues of the eigenmodes as a function of the distance traveled across the length of the nanowire implies exponential decay of the traveling SW (Fig. 3a). The propagating SW decays by dissipating its energy through damping process and the amplitude of the SW at any distance is typically modeled by^40^:

12 $$Y(l)={q}_{i}{e}^{-l/\varLambda }$$

13 $$\Lambda =\frac{2\gamma {A}_{ex}{k}_{x}}{\alpha {M}_{s}\pi f}$$

where Λ is the SW attenuation length, *γ* is the electron gyromagnetic ratio, *k*_*x*_ is the wave vector of the SW along the propagation direction, *α* is the damping constant. As aforementioned, the Gilbert damping constant *α* value is usually treated as a fixed value for a given sample. However, our results show that each eigenmode has a different decay trend, or attenuation length. By comparing Eqs (10) and (12), it is possible to define an eigen damping constant *α*_*i*_ to each of the eigenmodes:

14 $${\alpha }_{i}={\alpha }_{0}\frac{{k}_{xi}}{{k}_{x0}}\frac{\mathrm{ln}\,{m}_{i}}{\mathrm{ln}\,{m}_{0}}={\alpha }_{0}\frac{{\lambda }_{x0}}{{\lambda }_{xi}}\frac{\mathrm{ln}\,{m}_{i}}{\mathrm{ln}\,{m}_{0}}$$

where *k*_*xi*_ and *m*_*i*_ are the parallel wave vector along the wire direction and the eigenvalue of the *i*-th eigenmode for a given *f*, respectively. This is the central results of the present study. Here, each of the eigen damping constant *α*_*i*_ correspond to an eigenmode whose properties such as *k*_*xi*_ and *m*_*i*_ are obtained from the micromagnetic simulations and the transmission matrix calculations, which is different from the material-dependent damping constant^13–15^.

We calculated the corresponding eigenvalues of the first four eigenmodes for various nanowire width (Fig. 3b). The eigenvalues here are calculated for *l* = 200 nm away from the SW source. The results show that with wider nanowire, more eigenmodes are accessible. The results also show that when a new mode is just made accessible, it starts off with a lower eigenvalue than the preceding modes. For instance, at a nanowire width of 50 nm, the third eigenmode is accessible but it has a very low eigenvalue of ≈0.21 (Fig. 3b, red circle). However, as the nanowire width is increased, the third eigenmode is able to be accommodated well and it reaches a higher eigenvalue as compared to the 1^st^ and the 2^nd^ eigenmodes (when *w* > = 90 nm). It is observed to be a general trend and thus we may claim that for wider wire, higher modes have higher eigenvalue which means smaller eigen damping constant.

**Figure 4:**
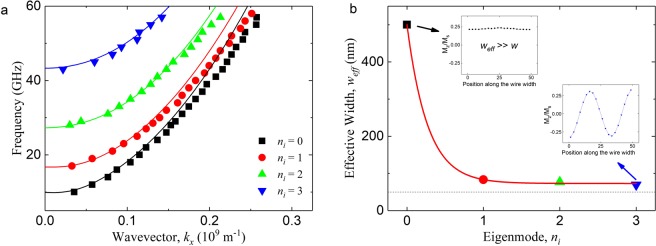
Dispersion relation of the eigenmodes. (**a**) Graph showing the dispersion relation of a 50 nm wide NiFe nanowire for various frequencies of the excitation field. The symbols represent the data obtained via micromagnetic simulations while the solid lines represent the analytical model. (**b**) The effective wire width, *w*_*eff*_, for the first four modes when the actual wire width, *w* (dashed black line), is 50 nm. Insets show the magnetization configurations of the *n*_*i*_ = 0 mode and the *n*_*i*_ = 3 mode. The red solid line is a guide for the eye.

We calculated the simulation results for the dispersion relation of the eigenmodes for a nanowire with width of 50 nm (Fig. 4a, symbols). The dispersion relation (Fig. 4a, solid line) for the various eigenmodes in the in-plane magnetized nanowire can be approximated by the analytic formula^39,41–46^:

15 $$f({k}_{x})=\frac{1}{2\pi }\sqrt{({\omega }_{M}({\lambda }^{2}{K}^{2}+{F}_{{k}_{x}}^{yy}))({\omega }_{M}({\lambda }^{2}{K}^{2}+{F}_{{k}_{x}}^{zz}))}$$

16 $${F}_{{k}_{x}}^{yy}=\frac{1}{2\pi \tilde{w}}{\int }_{-\infty }^{+\infty }{|\sigma |}^{2}\frac{{{k}_{y}}^{2}}{{{k}_{x}}^{2}+{{k}_{y}}^{2}}(1-(\frac{1-{e}^{(-\sqrt{({{k}_{x}}^{2}+{{k}_{y}}^{2})}t)}}{\sqrt{({{k}_{x}}^{2}+{{k}_{y}}^{2})}t}))d{k}_{y}$$

17 $${F}_{{k}_{x}}^{zz}=\frac{1}{2\pi \tilde{w}}{\int }_{-\infty }^{+\infty }{|\sigma |}^{2}(\frac{1-{e}^{(-\sqrt{({{k}_{x}}^{2}+{{k}_{y}}^{2})}t)}}{\sqrt{({{k}_{x}}^{2}+{{k}_{y}}^{2})}t})d{k}_{y}$$

18 $$\sigma =2[\frac{{k}_{y}\,\cos (\kappa w/2)\sin ({k}_{y}w/2)-\kappa \,\cos ({k}_{y}w/2)\sin (\kappa w/2)}{{{k}_{y}}^{2}-{\kappa }^{2}}]$$

19 $$\tilde{w}=\frac{w}{2}(1+{\rm{sinc}}(\kappa w))$$

where the term *F*_*kx*_^*yy*^ and *F*_*kx*_^*zz*^ account for dynamic demagnetization, λ = (2 A/μ_0_M_s_^2^)^1/2^ is the exchange length, ${\omega }_{M}=\gamma {\mu }_{0}{M}_{s}$, ${K}^{2}={{k}_{x}}^{2}+{\kappa }^{2}$, and $\kappa =\frac{({n}_{q}+1)\pi }{{w}_{eff,i}}$. The overall agreement is excellent when *k*_*x*_ < 0.15 × 10^9^ m^−1^. However, noticeable discrepancy can be seen for larger *k*_*x*_. This difference can be attributed to the fact that the nanowire in our simulation was only 5 nm thick, which gave us only a single layer of simulation cells along the nanowire thickness and thus reduced the accuracy of the micromagnetic simulations. Nevertheless, it did not affect our method of obtaining the transmission matrix nor it affects the general conclusion of the work. We calculated the effective width, *w*_*eff*,*i*_, of the first four eigenmodes for a nanowire with *w* = 50 nm (Fig. 4b) from the dispersion relation data (Fig. 4a). The result shows that the first eigenmode, *n*_*i*_ = 0, experiences the weakest edge pinning and thus perceives the largest *w*_*eff*_, while the subsequent modes experiences stronger pinning and perceive the effective width to be closer to the actual width of the nanowire.

**Figure 5:**
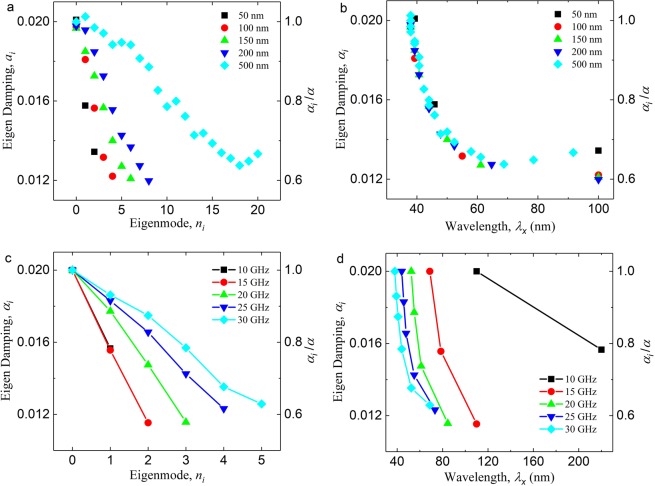
Eigen damping constants of the eigenmodes. (**a**) Graph showing the eigen damping constant for the various eigenmodes of nanowires with various widths. The applied frequency here is 30 GHz. The right axis shows the ratio between the eigen damping and the original damping constant of the material, *α*_*i*_*/α*. (**b**) Graph showing the eigen damping as function of the wavelength of the SW along the propagation direction for nanowires with various widths. (**c**) Graph showing the eigen damping as a function of the eigenmodes for various applied excitation frequency. (**d**) Graph showing the eigen damping as a function of the wavelength of the eigenmodes various applied excitation frequency. The wire width in (**c**,**d**) is 140 nm.

We calculated the eigen damping constant of the eigenmodes of the NiFe nanowire with various widths (Fig. 5a), which are obtained from the eigenvalues *q*_*i*_ from the micromagnetic simulations with Eq. (14), while keeping the frequency at *f* = 30 GHz. In general, for each wire, higher eigenmodes have lower eigen damping constant, i.e. *α*_*i*_ (*n*_*i*_ = 2, *w* = 50 nm) < *α*_*j*_ (*n*_*j*_ = 1, *w* = 50 nm), which leads to the higher amplitude as the SW travels along the nanowire. However, the same eigenmode will have larger eigen damping constant in a wider wire, i.e. *α*_*i*_ (*n*_*i*_ = 2, *w* = 500 nm) > *α*_*j*_ (*n*_*j*_ = 2, *w* = 50 nm). The wavelength versus eigen damping constant graph reveals the underlying relation between the eigenmodes and the eigen damping constants (Fig. 5b). The result shows that, when they are excited with the same frequency, eigenmodes with the same wavelength will share the same eigen damping constant, regardless of wire width and the mode number. Here, *λ*_*x*_ is the wavelength of the SW eigenmode along the propagation direction (*λ*_*x*_ = 2*π/k*_*x*_), and they are obtained from Fourier transform of propagating SW. The result also shows that the eigen damping constant continues to decrease as the wavelength is increased, at the smallest value of around *α* ≈ 0.012, which is equal to about 40% reduction as compared to the damping constant of the material, which is 0.02. In this example, the initial damping value of 0.02 for the simulations was chosen to shorten the overall calculation time for the transfer matrix. If we consider *α* = 0.012^47,48^ as the initial damping value for the simulations, we found that the eigen damping constant for higher eigenmodes can be reduced to *α* ≈ 0.006 (Supplementary Material III).

The eigen damping constant graph for various applied frequency (Fig. 5c) shows that increasing the applied frequency gives the same response as increasing the width of the nanowire, whereby more modes are allowed in the nanowire. In addition, increasing the applied frequency leads to a shift in the eigen damping vs. wavelength curve (Fig. 5d), which can be attributed to the inverse relationship between the wave vector and the applied frequency in Eq. (14).

## Discussion

The underlying physics of our finding, the eigen damping constant, is the interference of SW. For instance, a wave generated from a point-source in an infinite plane shall propagate without any interaction from any boundary. However, the wave from a point-source in a nanowire (or any nanostructure) shall encounter the boundaries of the wire and be reflected from the boundaries. Therefore, the interference between the original and the reflected waves is inevitable at the finite structure. Next, let us think about a plane wave excitation, which mathematically is a sum of point-source excitations in a nanowire. Most SW studies employed plane wave because it is the easiest way to generate a SW with a simple current wire. However, our results have shown that it is not the plane wave that has the highest transmission. As aforementioned, the higher SW eigenmodes keep the wave shape during traveling and they have higher eigenvalue, i.e. transmission rates (see Supplementary Material II and Supplementary Videos Eigen0-3.avi). It implies that the scattering at the edges are more effectively suppressed with higher eigenmode due to its complex wave shape, which leads to the lower damping constant. In addition, in Supplementary Material IV, we have also included a basic design of the SW injector that will be able to inject the eigenmodes that are shown in this work.

In conclusion, we have shown that it is possible to find the SW eigenmodes and eigen damping constants of any patterned nanostructure by developing a transmission matrix. The transmission matrix contains the information of how the SWs propagated at each point within the nanowire, and it is able to give us the eigenvalue of the eigenmodes, i.e. the transmission efficiency of the SW eigenmodes at any point along the length of the nanostructure. By employing our method to a nanowire with in-plane magnetization, we show that higher spinwave eigenmodes in general have higher eigenvalues, which means that they decay slower as they travel along the length of the nanowire. This also means that it is possible for us to assign unique damping constant to eigenmodes, with the higher modes having lower damping constant. Most importantly, the method that is presented in our work does not limit itself to the analysis of waves in ferromagnetic material, as we are mostly concerned with the interference capabilities of wave which is a trait shared by all kinds of wave in nature. Therefore, our method can be applied to any field of research who is concerned with either maximizing or minimizing the transfer of waves, such as the case of optical cloaking in optics^49,50^ and even seismic cloaking in geology^51,52^.

## Supplementary Information

Supplementary Information

Supplementary Video 1

Supplementary Video 2

Supplementary Video 3

Supplementary Video 4
