# Supplementary material for: Eigen damping constant of spin waves in ferromagnetic nanostructure
Source: Sci Rep. 2019 Sep 13;9:13226. doi: 10.1038/s41598-019-49872-w (PMC6744508; doi:10.1038/s41598-019-49872-w)
Supplement: Supplementary file 1 — Supplementary Information [file 41598_2019_49872_MOESM1_ESM.docx]

Manuscript Title: **Eigen damping constant of spin waves in ferromagnetic nanostructure**

Authors: Indra Purnama, Jung-Hwan Moon, and Chun-Yeol You

1. **Transmission Matrix building method**

a

b

d

e

c

Input

SW

Output

SW

x

y

z


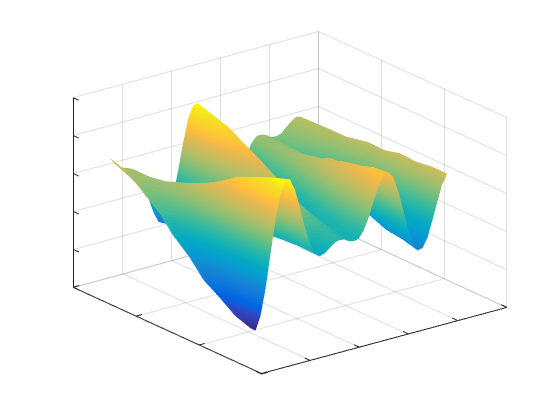

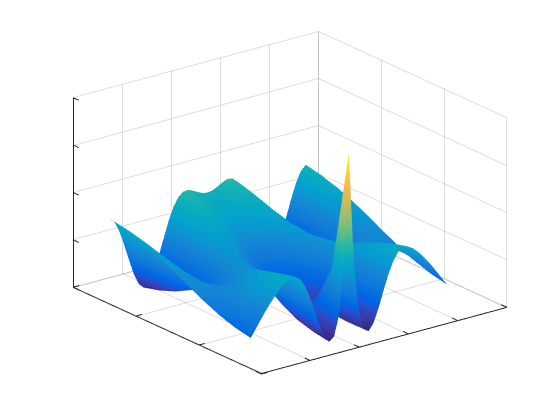


x

y

z

Point-sources

Point-outputs

Source 1

**Fig. S1. Spinwaves (SWs) from point-source 1. a,** Schematic of the first few steps in the process of building the transmission matrix. **b-c,** Visualization of the SWs that are observed at the point-sources and point-outputs when point-source 1 was excited was excited with *f* = 20 GHz. Point-source 1 was located at the edge of the nanowire. **d-e,** Plots of the magnetization at the point-sources and the point-outputs as functions of time.

a

d

b

c

e


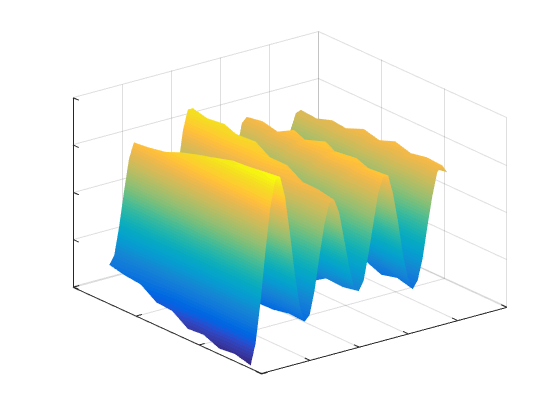

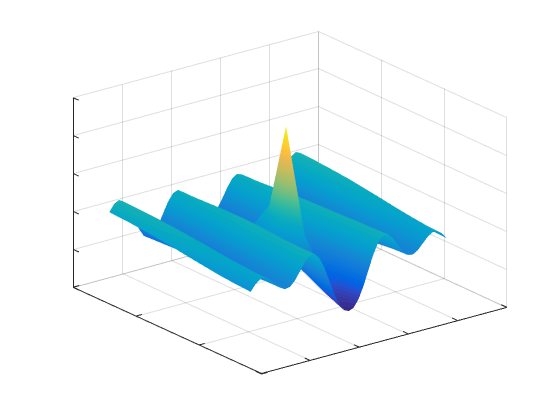


Input

SW

Output

SW

x

y

z

x

y

M_z_/M_s_

Point-sources

Point-outputs

Source 5

**Fig. S2. Spinwaves (SWs) from point-source 5. a,** Schematic of the first few steps in the process of building the transmission matrix. **b-c,** Visualization of the SWs that are observed at the point-sources and point-outputs when point-source 5 was excited with *f* = 20 GHz. Point-source 5 was located near the center of the nanowire. **d-e,** Plots of the magnetization at the point-sources and the point-outputs as functions of time.

The first step of the method is illustrated in Fig S1. As described in the manuscript, first we break up the spin wave (SW) at the source into several point-sources. To improve the accuracy of the calculation, it is recommended for each simulation cell to be designated as a point-source. Thus the total number of the point-source *N* is equal to = *w/c*, where *w* is the width of the nanowire and *c* is the simulation cell size. In this example, we simulated a 50 nm nanowire with simulation cell size of 5 nm, which gives us *N*=10. The simulations were performed using Mumax3 (*AIP Adv.* **4,** 107133 (2014)) micromagnetic simulators. For this example, we simulated a NiFe nanowire with thickness of 5 nm, the material parameters were chosen as following: saturation magnetization *M_s_* = 860 × 10^3^ A/m, exchange stiffness *A_ex_* = 1.3 × 10^-11^ J/m, and Gilbert damping = 0.02. We then proceeded to run a separate simulation for each point-source. In each simulation, external magnetic $H=H_{0}\sin(2\pi ft)$field is only applied to that specific point-source; *H_0_* = 100 mT while *f* is the oscillation frequency of the magnetic field. For instance, in the first simulation, only point-source 1 is excited, while in the fifth simulation, only point-source 5 is excited, etc. Fig. S1b shows the SW that is excited at the location of the point-sources during the application of external field to point-source 1. As can be seen from the image, at steady state, the magnetizations across the point-sources are all oscillating due to the exchange interaction even though the external magnetic field is only applied to point-source 1. Fig. S1d shows the magnetization evolution of the spins at the point-sources as a function of time, which reveals the differences between the amplitude and the phase of the oscillations at the point-sources. Fig. S1c and S1e show the SW after it has been transmitted to the location of the point-outputs, which show that the waves from each of the point-sources have undergone different amplitude attenuation as well as phase shifts when they arrive at the point-outputs. Similarly, Fig. S2 shows the SWs that are observed at the point-sources and the point-outputs when point-source 5 was excited

We then performed a fitting for each of the magnetization oscillation of the point-sources and point-outputs. Each magnetization oscillations are fitted to a sinusoidal function of $A\sin(\omega t+\varphi)$, where *A* is the amplitude, *f* is the oscillation frequency (which is the same as the oscillation frequency of the external magnetic field), and *ϕ* is the phase shift. As we have *N* = 10 point-sources, and *N*=10 point-outputs, we thus have 20 fitted data each of the simulation. The obtained numbers for the amplitudes and the phase shifts are then used to represent the SW at the source (*X*) and the output (*Y*). *X* and *Y* are column matrices where each component represents the oscillation at a specific point-source/ output:

$X_{i}\left( t \right)=\left( \begin{matrix} X_{1i}(t) \\ X_{2i}(t) \\ \begin{matrix} \vdots\\ X_{Ni}(t) \end{matrix} \end{matrix} \right)=\left( \begin{matrix} A_{1i}e^{\phi_{1i}} \\ A_{2i}e^{\phi_{2i}} \\ \begin{matrix} \vdots\\ A_{Ni}e^{\phi_{Ni}} \end{matrix} \end{matrix} \right)e^{i\omega t}$

$Y_{i}\left( t \right)=\left( \begin{matrix} Y_{1i}(t) \\ Y_{2i}(t) \\ \begin{matrix} \vdots\\ Y_{Ni}(t) \end{matrix} \end{matrix} \right)=\left( \begin{matrix} B_{1i}e^{\phi_{1i}} \\ B_{2i}e^{\phi_{2i}} \\ \begin{matrix} \vdots\\ B_{Ni}e^{\phi_{Ni}} \end{matrix} \end{matrix} \right)e^{i\omega t}$

Here, *i* denotes the point-source at which the external magnetic field was applied. *X_21_* means that it is the magnetization oscillation that is observed at the location of point-source 2 when external field is only applied to point-source 1. For instance, in Fig. S1, *i* = 1, and thus we have:

$H_{1}\left(t\right)=\left(\begin{matrix} H_{0}\sin\omega t \\ 0 \\ 0 \\ 0 \\ 0 \\ 0 \\ 0 \\ 0 \\ 0 \\ 0 \end{matrix}\right)$, $X_{1}\left(t\right)=\left(\begin{matrix} \text{-0.0054}+\text{0.0343i} \\ 0.0065+\text{0.0112i} \\ 0.0094+\text{0.0027i} \\ \text{0.0086-0.0013i} \\ \text{0.006-0.0027i} \\ \text{0.0024-0.0027i} \\ \text{-0.0014-0.0018i} \\ \text{-0.0049-0.0006i} \\ \text{-0.0076}+\text{0.0005i} \\ \text{-0.0094}+\text{0.0012i} \end{matrix}\right)\sin\omega t$, $Y_{1}\left(t\right)=\left(\begin{matrix} \text{-0.0006}-\text{0.0039i} \\ \text{-0.0008}-\text{0.0034i} \\ \text{-0.0009}-\text{0.0027i} \\ \text{-0.0012-0.0016i} \\ \text{-0.0014-0.0005i} \\ \text{-0.0016}+\text{0.0008i} \\ \text{-0.0018}+\text{0.0020i} \\ \text{-0.0020}+\text{0.0030i} \\ \text{-0.0020}+\text{0.0038i} \\ \text{-0.0021}+\text{0.0043i} \end{matrix}\right)\sin\omega t$

Where *X* and *Y* are normalized with respect to *M_s_*.

In the case of *i* = 5, as shown by Fig S2, we then have:

$H_{5}\left(t\right)=\left(\begin{matrix} 0 \\ 0 \\ 0 \\ 0 \\ H_{0}\sin\omega t \\ 0 \\ 0 \\ 0 \\ 0 \\ 0 \end{matrix}\right)$, $X_{5}\left(t\right)=\left(\begin{matrix} 0.0035-\text{0.0056i} \\ 0.0034-\text{0.0040i} \\ 0.0035-\text{0.0006i} \\ 0.0038+\text{0.0067i} \\ 0.0047+0\text{.0263i} \\ 0.0030+\text{0.0078i} \\ 0.0020+\text{0.0014i} \\ 0.0013-\text{0.0014i} \\ 0.0008-\text{0.0029i} \\ 0.0005-\text{0.0036i} \end{matrix}\right)\sin\omega t$, $Y_{5}\left(t\right)=\left(\begin{matrix} \text{-0.0014}+\text{0.0004i} \\ \text{-0.0013}+\text{0.0005i} \\ \text{-0.0013}+\text{0.0006i} \\ \text{-0.0013}+\text{0.0008i} \\ \text{-0.0012}+\text{0.0010i} \\ \text{-0.0011}+\text{0.0012i} \\ \text{-0.0011}+\text{0.0013i} \\ \text{-0.0009}+\text{0.0014i} \\ \text{-0.0009}+\text{0.0015i} \\ \text{-0.0008}+\text{0.0016i} \end{matrix}\right)\sin\omega t$

Mathematically, the two set of matrices are related to each other by:

$$Y_{1}=T\left(l\right)\cdot X_{1}$$

$$Y_{5}=T\left(l\right)\cdot X_{5}$$

Where *T* is a *N$\times$N* matrix transmission matrix that correlates the input SW matrix to the output SW matrix, and *l* is the distance between the input and the output. Again, in this example we have *N* = 10, and thus we can describe all of the simulation results by augmenting all of the *X_i_*’s and *Y_i_*’s into one equation:

$$Y\left( l \right)=\left( \left. \begin{matrix} Y_{11}(l) \\ \vdots\\ Y_{N1}(l) \end{matrix} \right|\left. \begin{matrix} Y_{12}(l) \\ \vdots\\ Y_{N2}(l) \end{matrix} \right|\begin{matrix} Y_{1N}(l) \\ \vdots\\ Y_{NN}(l) \end{matrix} \right)=T\left( l \right)\cdot X=T\left( l \right)\cdot\left( \left. \begin{matrix} X_{11}(l) \\ \vdots\\ X_{N1}(l) \end{matrix} \right|\left. \begin{matrix} X_{12}(l) \\ \vdots\\ X_{N2}(l) \end{matrix} \right|\begin{matrix} X_{1N}(l) \\ \vdots\\ X_{NN}(l) \end{matrix} \right)$$

If we compare Fig. S1d to S1e, as well as Fig S2d to S2e, it is clear that the SW gained different amount of phase shift and amplitude attenuation across its wave profile for each of the two cases. For instance, initially the magnetization oscillation at point-source 1 has a phase difference of ≈ π/2 with respect to the oscillation at point-source 10. However, at the outputs, the magnetization oscillation of point-output 1 now has a phase difference of ≈ π. Therefore, we can expect that the off-diagonal components of *T* are non-zero in general. Additionally, it is important to note that *Y* and *X* are to be built by using the data from all *N* simulations, i.e. Y= (Y_1_| Y_2_|…|Y_N_). In this method, each simulation refers to the case where the field is excited at specific point-source/ simulation cell. So Y1 and X1 will be obtained from the simulation where point-source 1 is excited, while Y3 and X3 are obtained from point-source 3 excitation, and so on.

In Fig. S3 we compare the superposition of the magnetization oscillations at the point-outputs from all the *N* simulations, to a standard plane wave. Here, Output1 in Fig. S3a refers to the case of Y_1_=Y_11_+Y_12_+Y_13_+Y_14_+Y_15_+Y_16_+Y_17_+Y_18_+Y_19_+Y_110_, while Output1 in Fig. S3b is the magnetization obtained from the plane wave excitation. We can see that the magnetization oscillations at the different outputs after superposition are now in the same phase and relatively same amplitude, much like to a plane wave.

b

a

**Fig. S3. Linear superposition of the SWs generated by the point-sources a,** The superposition of the magnetization oscillations that occur at the point-outputs for all N=10 simulations. **b,** The magnetization oscillations at the point-outputs from a plane wave excitation.

The transmission matrix, *T*, can then be built by multiplicating *Y* with the inverse of *X*:

$$T\left(l\right)=Y\left(l\right)\cdot\left(X\right)^{-1}$$

Note that the time dependency (sin *2πf*t) has cancelled out from the equation, and therefore we only need the amplitudes as well as the phase shifts of the magnetizations to make the transmission matrix. After we obtain *T*, we can then find its eigenvalues as well as eigenvectors associated to them. However, also note that *T* is inherently a function of *f* and distance *l,* or in other words, to find the eigenvectors at different frequency and distance *l*, we have to do the simulations again and extract the data again. The eigenvectors are sets of column matrices with components that describe the oscillation amplitudes as well as the phase shifts that have to be excited at the sources in order to create the SW eigenmodes of the nanostructure. Below is an example of *T* obtained from the set of 10 simulations which include the data shown in Fig. S1 and Fig. S2.

Column 1 to 5:

$$T=\left[\begin{matrix} \text{-0.0534 - 0.0189i} \\ \text{-0.0474 - 0.0122i} \\ \text{-0.0379 - 0.0024i} \\ \text{-0.0250 }+\text{ 0.0131i} \\ \text{-0.0110 }+\text{ 0.0286i} \\ \text{0.0062 }+\text{ 0.0458i} \\ \text{0.0221 }+\text{ 0.0612i} \\ \text{0.0347 }+\text{ 0.0738i} \\ \text{0.0453 }+\text{ 0.0827i} \\ \text{0.0515 }+\text{ 0.0895i} \end{matrix}\begin{matrix} \text{-0.0483 - 0.0124i} & \text{-0.0395 - 0.0005i} & \text{-0.0261 }+\text{ 0.0153i} & \text{-0.0102 }+\text{ 0.0325i} & & & & & & \\ \text{-0.0427 - 0.0061i} & \text{-0.0345 }+\text{ 0.0046i} & \text{-0.0242 }+\text{ 0.0177i} & \text{-0.0090 }+\text{ 0.0333i} & & & & & & \\ \text{-0.0341 }+\text{ 0.0030i} & \text{-0.0284 }+\text{ 0.0120i} & \text{-0.0176 }+\text{ 0.0238i} & \text{-0.0080 }+\text{ 0.0361i} & & & & & & \\ \text{-0.0222 }+\text{ 0.0170i} & \text{-0.0179 }+\text{ 0.0232i} & \text{-0.0140 }+\text{ 0.0311i} & \text{-0.0049 }+\text{ 0.0392i} & & & & & & \\ \text{-0.0092 }+\text{ 0.0307i} & \text{-0.0080 }+\text{ 0.0346i} & \text{-0.0053 }+\text{ 0.0385i} & \text{-0.0036 }+\text{ 0.0428i} & & & & & & \\ \text{0.0050 }+\text{ 0.0467i} & \text{0.0034 }+\text{ 0.0474i} & \text{0.0019 }+\text{ 0.0466i} & \text{0.0001 }+\text{ 0.0447i} & & & & & & \\ \text{0.0196 }+\text{ 0.0595i} & \text{0.0155 }+\text{ 0.0574i} & \text{0.0091 }+\text{ 0.0544i} & \text{0.0019 }+\text{ 0.0476i} & & & & & & \\ \text{0.0307 }+\text{ 0.0713i} & \text{0.0242 }+\text{ 0.0669i} & \text{0.0155 }+\text{ 0.0578i} & \text{0.0050 }+\text{ 0.0477i} & & & & & & \\ \text{0.0402 }+\text{ 0.0783i} & \text{0.0321 }+\text{ 0.0719i} & \text{0.0204 }+\text{ 0.0628i} & \text{0.0063 }+\text{ 0.0491i} & & & & & & \\ \text{0.0457 }+\text{ 0.0845i} & \text{0.0365 }+\text{ 0.0772i} & \text{0.0234 }+\text{ 0.0651i} & \text{0.0077 }+\text{ 0.0499i} & & & & & & \end{matrix}\right.$$

Column 6 to 10:

$$\left.\begin{matrix} \text{0.0066 }+\text{ 0.0501i} & \text{0.0222 }+\text{ 0.0659i} & \text{0.0351 }+\text{ 0.0782i} & \text{0.0443 }+\text{ 0.0857i} & \text{0.0500 }+\text{ 0.0901i} \\ \text{0.0054 }+\text{ 0.0492i} & \text{0.0195 }+\text{ 0.0633i} & \text{0.0312 }+\text{ 0.0725i} & \text{0.0392 }+\text{ 0.0791i} & \text{0.0442 }+\text{ 0.0831i} \\ \text{0.0046 }+\text{ 0.0478i} & \text{0.0152 }+\text{ 0.0579i} & \text{0.0238 }+\text{ 0.0670i} & \text{0.0304 }+\text{ 0.0714i} & \text{0.0343 }+\text{ 0.0739i} \\ \text{0.0020 }+\text{ 0.0477i} & \text{0.0094 }+\text{ 0.0543i} & \text{0.0158 }+\text{ 0.0571i} & \text{0.0199 }+\text{ 0.0592i} & \text{0.0223 }+\text{ 0.0611i} \\ \text{0.0003 }+\text{ 0.0447i} & \text{0.0024 }+\text{ 0.0466i} & \text{0.0040 }+\text{ 0.0474i} & \text{0.0056 }+\text{ 0.0468i} & \text{0.0069 }+\text{ 0.0458i} \\ \text{-0.0036 }+\text{ 0.0429i} & \text{-0.0052 }+\text{ 0.0388i} & \text{-0.0078 }+\text{ 0.0351i} & \text{-0.0091 }+\text{ 0.0313i} & \text{-0.0100 }+\text{ 0.0292i} \\ \text{-0.0048 }+\text{ 0.0392i} & \text{-0.0140 }+\text{ 0.0314i} & \text{-0.0179 }+\text{ 0.0235i} & \text{-0.0223 }+\text{ 0.0172i} & \text{-0.0245 }+\text{ 0.0137i} \\ \text{-0.0079}+\text{ 0.0362i} & \text{-0.0176 }+\text{ 0.0239i} & \text{-0.0283 }+\text{ 0.0121i} & \text{-0.0340 }+\text{ 0.0033i} & \text{-0.0380 - 0.0022i} \\ \text{-0.0090 }+\text{ 0.0336i} & \text{-0.0241 }+\text{ 0.0177i} & \text{-0.0342 }+\text{ 0.0049i} & \text{-0.0425 - 0.0055i} & \text{-0.0480 - 0.0123i} \\ \text{-0.0104 }+\text{ 0.0328i} & \text{-0.0258 }+\text{ 0.0153i} & \text{-0.0391 }+\text{ 0.0000i} & \text{-0.0481 - 0.0115i} & \text{-0.0543 - 0.0192i} \end{matrix}\right]$$

1. **Eigenmodes of the nanostructure**

After we have obtained the transmission matrix, computational calculation will give us its eigen vectors, i.e. the SW eigenmodes for of our nanostructure. For the above *T,* which was obtained at *f* = 20 GHz, we will obtain the *n_i_* = 0 and the *n_i_* = 1 eigenmode:

$$X_{ni=0}=\left(\begin{matrix} \text{0.3090 + 0.0002i} \\ \text{0.3080 - 0.0005i} \\ \text{0.3128 - 0.0011i} \\ \text{0.3230 - 0.0038i} \\ \text{0.3259 - 0.0011i} \\ 0.3278 \\ \text{0.3251 - 0.0025i} \\ \text{0.3137 - 0.0016i} \\ \text{0.3078 - 0.0030i} \\ \text{0.3081 - 0.0033i} \end{matrix}\right)$$

$$X_{ni=1}=\left(\begin{matrix} \text{-0.4486 - 0.0057i} \\ \text{-0.3944 - 0.0020i} \\ \text{-0.3126 - 0.0037i} \\ \text{-0.2004+ 0.0022i} \\ \text{-0.0732 + 0.0009i} \\ \text{0.0690 + 0.0018i} \\ \text{0.1991 - 0.0003i} \\ \text{0.3125 + 0.0032i} \\ \text{0.3949 - 0.0016i} \\ 0.4494 \end{matrix}\right)$$

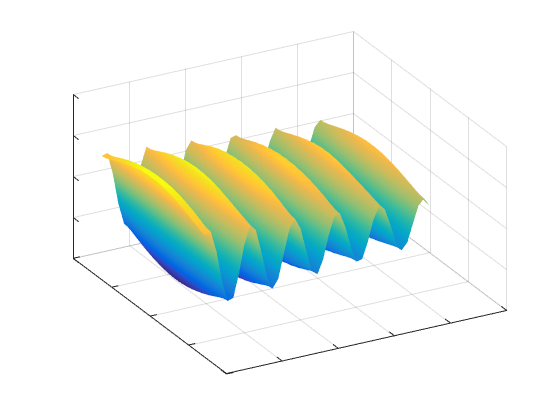

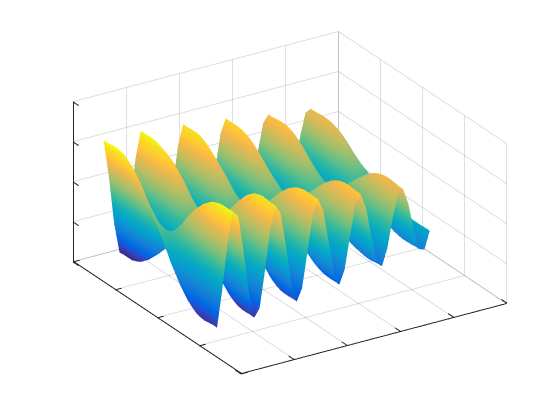


(b)

(a)

**Fig. S4. Visual representation of the SW eigenmodes. a,** SW eigenmode *n_i_* = 0 and **b,** SW eigenmode *n_i_* = 1 .

One characteristic of an eigenmode is that it retains its shape during its propagation along the nanowire. In Fig. S5, we compare the magnetization configuration of the SWs at the input and the output for the case of eigenmode *n_i_* = 3 and eigenmode *n_i_* = 4 obtained by our method to show that they are able to retain their relative amplitude and phase shift and that they are both eigenmodes of the nanowire. We also submitted separate video files (Eigen0.avi-Eigen3.avi) to show the propagation of eigenmode with *n_i_* = 0-3, respectively. In the Supplementary videos, the SW eigenmodes are shown to travel from one side of the video to the other side without any change in the shape of the SWs, which further confirm that they are the eigenmodes of the system.

(b)

(a)

**Fig. S5. Magnetization configuration of the SWs at the input and output.** **a,** SW eigenmode *n_i_* = 3 and **b,** SW eigenmode *n_i_* = 4 at the input and the output.

1. **Micromagnetic simulations supporting data**

In general, we have shown that the transmission matrix method is able to find the eigenmodes of a magnetic system together with their eigenvalues as well as eigen damping constants. In our manuscript, the results that are shown were obtained using simulation cellsize of 5 nm × 5 nm × 5 nm and initial damping *α* = 0.02 in order to reduce the calculation time. In Fig. S6a, we show the simulation results of a 200 nm wide nanowire with initial damping constant of *α* = 0.012. The results show that higher eigenmodes have smaller eigen damping constants, which is the same behavior as the simulations that were run with initial damping of *α* = 0.02. In Fig. S6b, we show the simulation results of a 140 nm wide nanowire with simulation cell size of 2.5 nm × 2.5 nm × 2.5 nm. We see that the eigenvalues show the same behavior as simulations with cell size of 2.5 nm × 2.5 nm × 2.5 nm, which is a gradual increase in the eigenvalue followed by a sharp drop for eigenmodes that cannot be accommodated by the narrow nanowire. The results shown here imply that the relation between the higher eigenmodes and the decreasing eigendamping constants does not depend strongly on the initial simulation parameters. And thus, the simulation parameters that are used in the manuscript is adequate to demonstrate the transmission matrix calculation method while still maintaining reasonable calculation time.

(b)

(a)

**Fig. S6 a,** Micromagnetic simulations with smaller initial damping constant. The nanowire width here is 200 nm. Red solid line is a guide for the eye. The eigendamping constant is shown to be reduced for higher eigenmodes. **b**, Micromagnetic simulations with smaller cell size. The nanowire width here is 140 nm. The eigenvalue of the modes is shown to increase for the modes that can be accommodated by the narrow nanowire while it drops sharply for the modes that cannot be accommodated.

In the simulations, the thickness of 5 nm was chosen for the nanowire to expedite the simulation time as well as the following transmission matrix calculation time. In the simulation, the magnetic field for the spinwave excitation at the point source is generated across the nanowire thickness. For thin nanowire (5-10 nm), this means that the magnetization oscillation is the same across the layers in the micromagnetic simulation. The feasibility of generating spinwaves in a thin permalloy has been demonstrated by Silvani *et al^53^*.

The results of applying the transmission matrix method to nanowires with various saturation magnetization (*M_s_*) and exchange constant (*A_ex_*) are shown below. The results show that increasing *M_s_* or decreasing *A_ex_* will result in the decrease of the eigenvalue of the eigenmodes. However, the overall behavior and relationship between the eigenvalues and the eigenmodes are not changed. Once again, this results further shows the robustness of the transmission matrix method.

a

b

**Fig. S7 a,** Eigenvalues of the in-plane magnetized nanowire with various material parameter. b, The eigenmodes of the nanowire with various material parameters.

1. **Higher-mode SW Injector**

SWs are typically injected into a nanowire by applying current to a straight conductor line that is patterned orthogonal to the nanowire. However, only the standard plane wave is able to be injected by this method. To inject the higher-mode eigen SWs, we would need an injector that is able to create magnetic fields with opposite phases along the widths of the nanowire. A possible design for such SW injector is presented below:

**Fig. S8. Zig-zag injector for SW eigenmode.** Design of the SW injector for eigenmode *n_i_* = 1. The gold zig-zag line is the SW injector while the big blue bar is the SW medium.

The above design is a SW injector for eigenmode 1, i.e. *n_i_* = 1. In this design, half of the injector is shifted forward along the nanowire. This is done so that the magnetic fields (with opposite phase) from the two sides of the injector are aligned together along the width of the nanowire. By doing so, the SW that is created in the nanowire will have opposite phase to each other, which is the main feature of eigenmode 1. Fig. S9 shows the magnetic field that is created around the SW injector. In this example the magnetic nanowire has a width of 500 nm.


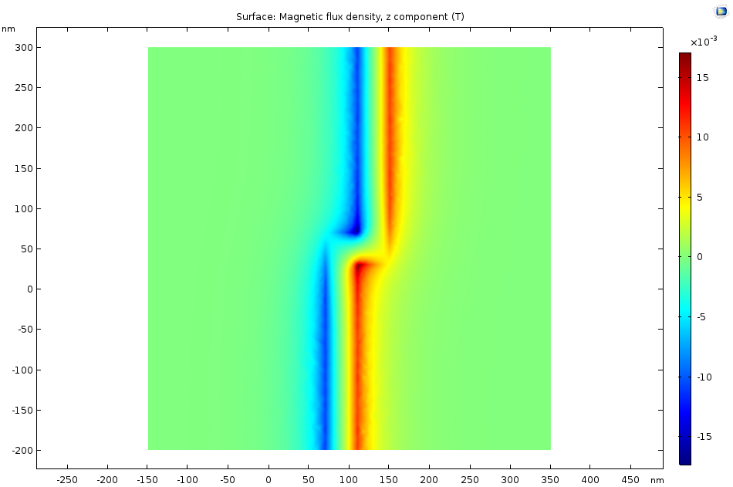


a

b

**Fig. S9. Magnetic field created by the zig-zag injector. a,** Top-view of the magnetic field created by the SW injector. The highlighted area refers to the center of the injector where the magnetic fields are of opposite phase along the width of the nanowire. The simulation here was performed using COMSOL. The injector line has a width of 40 nm and thickness of 10 nm. The chosen material parameter for the injector line corresponds to copper, and the injector line is injected with 10μA current. **b,** Plot showing the strength of the magnetic field along the centerpiece of the injector.

For higher eigenmode, the injector can be modified by increasing the zig-zag repetition.

**Fig. S10. Magnetic field created by the zig-zag injector.** Design of the SW injector for eigenmode *n_i_* = 2.

**Supplementary References (numbering continued from main manuscript)**

53. Silvani, R., Kostylev, M., Adeyeye, A. O. & Gubbiotti, G. Spin wave filtering and guiding in Permalloy/iron nanowires. *J. Magn. Magn. Mater.* **450**, 51–59 (2018).
